# A Guideline to Univariate Statistical Analysis for LC/MS-Based Untargeted Metabolomics-Derived Data

**DOI:** 10.3390/metabo2040775

**Published:** 2012-10-18

**Authors:** Maria Vinaixa, Sara Samino, Isabel Saez, Jordi Duran, Joan J. Guinovart, Oscar Yanes

**Affiliations:** 1 Metabolomics Platform, Campus Sescelades, Edifici N2, Rovira i Virgili University, Tarragona 43007, Spain; Email: sara.samino@urv.cat (S.S.); 2 Spanish Biomedical Research Center in Diabetes and Associated Metabolic Disorders (CIBERDEM), Passeig Bonanova 69, Barcelona 08017, Spain; Email: jordi.duran@irbbarcelona.org (J.D.); guinovart@irbbarcelona.org (J.J.G.); 3 Institut d’Investigació Biomédica Pere Virgili (IISPV), C/Sant Llorenç, 21, Reus 43201, Spain; 4 Institute for Research in Biomedicine (IRB Barcelona), Barcelona 08028, Spain; Email: isabel.saez@irbbarcelona.org (I.S.); 5 Department of Biochemistry and Molecular Biology, University of Barcelona, Barcelona 08028, Spain

**Keywords:** univariate, metabolomics, mass spectrometry.

## Abstract

Several metabolomic software programs provide methods for peak picking, retention time alignment and quantification of metabolite features in LC/MS-based metabolomics. Statistical analysis, however, is needed in order to discover those features significantly altered between samples. By comparing the retention time and MS/MS data of a model compound to that from the altered feature of interest in the research sample, metabolites can be then unequivocally identified. This paper reports on a comprehensive overview of a workflow for statistical analysis to rank relevant metabolite features that will be selected for further MS/MS experiments. We focus on univariate data analysis applied in parallel on all detected features. Characteristics and challenges of this analysis are discussed and illustrated using four different real LC/MS untargeted metabolomic datasets. We demonstrate the influence of considering or violating mathematical assumptions on which univariate statistical test rely, using high-dimensional LC/MS datasets. Issues in data analysis such as determination of sample size, analytical variation, assumption of normality and homocedasticity, or correction for multiple testing are discussed and illustrated in the context of our four untargeted LC/MS working examples.

## 1. Introduction

The comprehensive detection and quantification of metabolites in biological systems, coined as ‘metabolomics’, offers a new approach to interrogate mechanistic biochemistry related to natural processes such as health and disease. Recent developments in mass spectrometry (MS) and nuclear magnetic resonance (NMR) have been crucial to facilitate the global analysis of metabolites. The examination of metabolites, however, commonly follows two strategies: (i) targeted metabolomics, driven by a specific biochemical question or hypothesis in which a set of metabolites related to one or more pathways are defined, or (ii) untargeted metabolomics: driven by an unbiased approach (*i.e.*, non-hypothesis) in which as many metabolites as possible are measured and compared between samples [1]. The latter is comprehensive in scope and outputs complex data sets, particularly by using LC/MS-based methods. Thousands of so called metabolite features (*i.e.*, peaks corresponding to individual ions with a unique mass-to-charge ratio and a unique retention time or mzRT features from now on) can be routinely detected in biological samples. In addition, each mzRT feature in the dataset is associated with an intensity value (or area under the peak), which indicates its relative abundance in the sample. Overall, this complexity imposes the implementation of metabolomic softwares such as XCMS [2], MZmine [3] or Metalign [4] that can provide automatic methods for peak picking, retention time alignment to correct experimental drifts in instrumentation, and relative quantification. As a result, the identification of mzRT features that are differentially altered between sample groups has become a relatively automated process. However, the identification and quantization of a “metabolite feature” does not necessary translate into a metabolite entity. LC/MS metabolomic data presents high redundancy because of the recurrent detection of adducts (Na+, K+, NH3, etc), isotopes, or doubly charged ions that greatly inflate the number of detected peaks. Several recently launched open-source algorithms such as CAMERA [5] or AStream [6], and commercially available software such as Mass Hunter (Agilent Technologies) or Sieve (Thermo Scientific), are capable of filtering redundancy by annotating isotopes and adduct peaks, and the resulting accurate compound mass (*i.e.*, molecular ion) can be searched in metabolite databases such as METLIN, HMDB or KEGG. Database matching represents only a putative metabolite assignment that must be confirmed by comparing the retention time and/or MS/MS data of a model pure compound to that from the feature of interest in the research sample. These additional analyses are time consuming and represent the rate-limiting step of the untargeted metabolomic workflow. Consequently, it is essential to prioritize the list of mzRT features from the raw data that will be subsequently identified by RT and/or MS/MS comparison. Relevant mzRT features for MS/MS identification are typically selected based on statistics criteria, either by multivariate data analysis or multiple independent univariate tests. 

The intrinsic nature of biological processes and LC/MS-derived datasets is undoubtedly multivariate since it involves observation and analysis of more than one variable at a time. Consequently, the majority of metabolomics studies make use of multivariate models to report their main findings. Despite the conferred utility, powerfulness and versatility of multivariate models, their performance might be fraught by the high-dimensionality of such datasets due to the so-called ‘curse of dimensionality’ problem. Curse of dimensionality arises when datasets contain too much sparse data in terms of the number of input variables. This causes, in a given sample size, a maximum number of variables above which the performance of our multivariate model will degrade rather than improve. Hence, attempting to make the model conform too closely to this data (*i.e.*, considering too many variables in our multivariate model) can introduce substantial errors and reduce its predictive power (*i.e.*, overfitting). Therefore, using multivariate models require intensive validation work. Overall, multivariate data analysis is far from the scope of this paper and excellent reviews on multivariate tools for metabolomics can be found elsewhere [7,8]. On the other hand, data analysis can also be approached from a univariate perspective using traditional statistical methods that consider only one variable at a time [9]. The implementation of multivariate and univariate data analysis is not mutually exclusive and in fact, we strongly recommend their combined use to maximize the extraction of relevant information from metabolomic datasets [10,11]. Univariate methods are sometimes used in combination with multivariate models as a filter to retain those potentially “information-rich” mzRT features [12]. Then, the number of mzRT features considered in the multivariate model is significantly reduced down to those showing statistical significance in previous univariate tests (e.g., p-value < 0.05). On the other hand, there are multiple reported metabolomics works using univariate tests applied in parallel across all the detected mzRT features to report their main findings. It should be note that this approach overlooks correlations within mzRT features and therefore information about correlated trends is not retained. In addition, applying multiple univariate tests in parallel to multivariate datasets involves the acceptance of mathematical pre-requisites and certain consequences such as the particular distributions of variables (e.g., normality) and increased risk of false positive results, respectively. Many researchers often ignore these issues when analyzing untargeted metabolomic datasets using univariate methods, which eventually can compromise their results. 

This paper aims to investigate the impact of univariate statistical issues on LC/MS-based metabolomic experiments, particularly in small, focused studies (e.g., small clinical trials or animal studies). To this end, here we explore the nature of four real and independent datasets, evaluate the challenges and limitations of executing multiple univariate tests and illustrate available shortcuts. Note that we do not aim at writing a conventional statistical paper. Instead, our goal is to offer a practical guide with resources to overcome the challenges of multiple univariate analysis for untargeted metabolomic data. All methods described in this paper are based on scripts programmed either in MATLAB™ (Mathworks, Natick, MA) or R [13] . 

## 2. Properties of LC-MS Untargeted Datasets: High-Dimensional and Multicolinear

Basic information about the four real untargeted metabolomics LC-MS-based working examples is summarized in Table 1. These examples do not resemble ideal datasets described in basic statistical textbooks, and illustrates the challenges of real-life metabolomic experiments. Working examples constitute retinas, serum and neuronal cell cultures under different experimental conditions (e.g., KO *vs*. WT; normoxia *vs*. hypoxia; treated *vs*. untreated) analyzed by LC-qTOF MS. Data were processed using the XCMS software to detect and align features, and thousands of features were generated from these biological samples. Each mzRT feature corresponds to a detected ion with a unique mass-to-charge ratio, retention time and raw intensity (or area). For example, each sample in example #3 exists in a space defined by 9877 variables or mzRT features. The four examples illustrate the high-dimensionality of untargeted LC-MS datasets in which the number of features or variables largely exceeds the number of samples. The rather limited number of individuals or samples per group is a common trait of metabolomic studies devoted to understand cellular metabolism [14,15,16]. When working with animal models of disease, for instance, this limitation is typically imposed by ethical and economical restrictions.

**Table 1:** Summary of working examples obtained from LC-MS untargeted metabolomic experiments. Further experimental details and methods can be obtained from references. (KO=Knock-Out; WT=Wild-Type).

	Biofluid/Tissue	Sample groups	# samples /group	# XCMS variables	System	Reference
Example #1	Retina	KO	11	4581	LC/ESI-QTOF	[17]
		WT	11			
Example #2	Retina	Hypoxia	12	8146	LC/ESI-QTOF	[16]
		Normoxia	13			
Example #3	Serum	Untreated	12	9877	LC/ESI-TOF	[18]
		Treated	12			
Example #4	Neuronal cell cultures	KO	15	8221	LC/ESI-QTOF	unpublished data
		WT	11			

Additionally, a second attribute of untargeted LC-MS metabolomic datasets is that they enclose multiple correlations among mzRT features (*i.e.*, multicollinearity) [19]. Each metabolite produces more than one mzRT feature that result from isotopic distributions, potential adducts, and in-source fragmentation. Moreover, the evident biochemical interrelation among metabolites may also contribute to the multicollinearity. Namely, many metabolites participate in inter-connected enzymatic reactions and pathways (e.g., substrate and product; cofactors) and regulate enzymatic reactions (e.g., feed-back inhibition). Altogether, untargeted LC-MS metabolomics datasets are highly-dimensional and multicorrelated. 

## 3. Sample Size Calculation in LC-MS Untargeted Metabolomics Studies

The number of subjects per group (*i.e.*, sample size) is an important aspect to be determined during the experimental design of the study. A low sample size may lead to a lack of precision, which may fail to provide reliable clues about the biological question under investigation. In contrast, an unnecessarily high sample size may lead to a waste of resources for minimal information gain. Thus it is not surprising that funding agencies require power/sample size calculations in their grant proposals. However, choosing the appropriate sample size for high-throughput approaches involving multivariate data is complicated. According to Hendriks *et al.* [8], there is currently nothing available for *a priori* sample size estimation of highly collinear multivariate data. 

Traditional univariate sample size determination is based in the concept of power analysis. Power, or the sensitivity of the test, is defined as 1-β, being β the chance of a false negative or Type II error in hypothesis testing. A Type II error is produced when a variable is claimed to not be significant when in fact it is. Therefore, power can be defined as the probability of a statistical test to allow detection of significant differences above a certain confidence. Classical power analysis to determine minimum sample size for a given variable (*i.e.*, metabolite) requires the estimation of population means and standard deviations and effect sizes. However, for high-dimensional data such estimates need to be redefined. Average power is used instead of power, significance level needs to take multiple testing into account and both effect sizes and variances take multiple values. Ferreira *et al*. [20,21] extended the concept of power analysis to high-dimensional data using univariate approaches in combination with multiple testing corrections. They used the entire set of test statistics from microarray pilot data to estimate the effect size distribution, power and minimal sample size. This method have been recently generalized and adapted by van Iterson *et al.* [22] as a part of the BioConductor package SSPA. Recall that using this method, data is treated as a set of multiple univariate responses and correlations between variables are ignored. On the other hand, this method was designed to guide experimental design decisions based on previously acquired pilot data. However, how realistic is to perform a pilot untargeted metabolomics study to determine minimum sample size? In practice, ethical and economical restrictions mainly determine the number of samples (*i.e.*, animals) for each group. 

**Figure 1:**
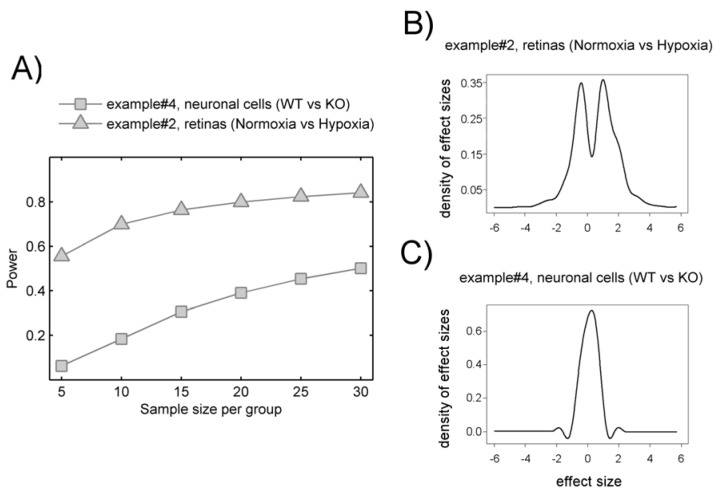
(A) Power curves for example #2 (∆) and example #4 (□) with sample size on the x-axis and estimated power using 5% FDR on the y-axis. Estimated densities of effect sizes for example #4 (B) and example #2 (C) with the standardized effect size on x-axis and estimated densities on the y-axis. Bimodal densities as in example #2 reflect more pronounced effects.

Although we recognize the limitations and controversy of post-hoc power analysis, for illustrative purposes we used SSPA to estimate effect sizes and perform power calculations of our untargeted metabolomics data. Figure 1A show a comparison of example #2 and example #4 estimated power values considering up to 30 samples per group. Considering example #2, a 70% power to detect hypoxia-induced metabolic differences was obtained with 10 retinas per groups. This power was associated with a markedly bimodal density of effects sizes (Figure 1B) indicating significant hypoxia-induced metabolic variation. The density of effects sizes describes the effects observed in the data. Usually, a bimodal density is observed when the studied effect induces significant differences. In contrast, even considering up to 30 samples per group we end-up with low power to detect KO-induced differences in example#4 (Figure 1C). This indicates that KO-induced effects are scarcely reflected in our metabolomics data as represented by its unimodal densities of effects sizes Accordingly, we would estimate a minimum of ten samples per group (n = 10) as the easiest way to boost the statistical power of univariate statistical tests when true metabolic differences exist between two groups (e.g., example #2 comparing normoxia *vs*. hypoxia). This post-hoc calculation of the statistical power and sample size could be taken as a rough estimation for follow-up validation studies using triple quadrupole (QqQ) instrumentation.

## 4. Handling Analytical Variation

The first issue that must be resolved before considering any univariate statistical test on LC/MS untargeted metabolomic data is analytical variation. Most common sources of analytical variation in LC-MS experiments are due to sample preparation, instrumental drifts caused by chromatographic columns and MS detectors, and errors caused in data processing [23]. 

The ideal method to examine analytical variation is to analyze quality control (QC) samples, which will provide robust quality assurance of each detected mzRT feature [24]. To this end, QC samples should be prepared by pooling aliquots of each individual sample and analyze them periodically throughout the sample work list. The performance of the analytical platform for each detected mzRT feature in real samples can be assessed by calculating the relative standard deviation of these features on pooled samples (CV_QC_) according to formula Equation (1), where S and X are respectively the standard deviation and the mean of each individual feature detected across the QC samples:


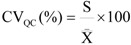
(1)

Likewise, the relative standard deviation of these features on study samples (CV_T_) can be defined according to formula Equation (2), where S and X are the standard deviation and mean respectively calculated for each mzRT feature across all study samples in the dataset.


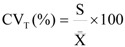
(2)

**Figure 2:**
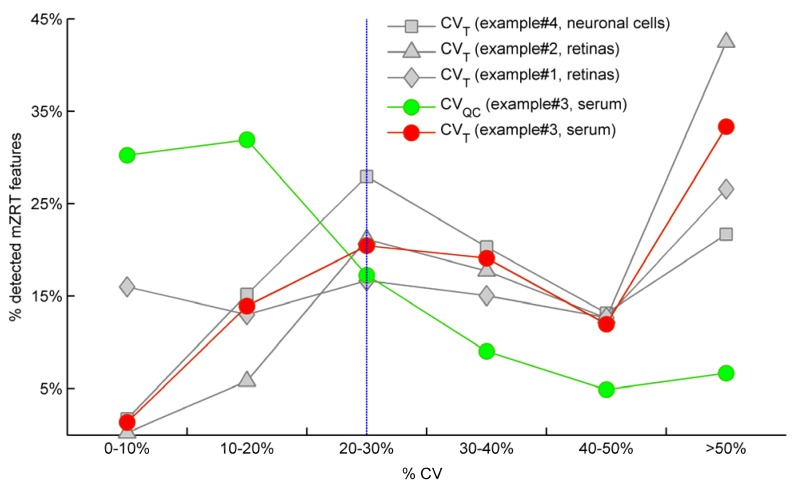
Comparison for our four working examples of the mzRT relative standard deviation (CV) frequency distributions calculated either across all the samples (CV_T_) or across QC samples (CV_QC_). Grey spots represent CV_T_ for examples #1(◊), #2 (∆) and #4 (□) respectively. Green and red circles represent CV_QC_ and CV_T_ respectively for example #3. Blue line represents 20% CV_T_ cut-off threshold established when QC samples are not available.

The variation of QC samples around their mean (CV_QC_) is expected to be low since they are replicates of the same pooled samples. Therefore Dunn *et al*. [24] have established a quality criteria by which any peak that presents a CV_QC_ > 20% is removed from the dataset and thus ignored in subsequent univariate data analyses. Red and green spots in Figure 2 illustrate the CV_T_ and CV_QC_ frequencies distributions respectively for example #3 in which QC samples were measured. As expected, the highest percentage of mzRT features detected across QC samples present the lowest variation in terms of CV_QC_ (green line). Conversely, the highest percentage of the mzRT features detected across the study samples holds the highest variation in terms of CV_T_ (red line). Notice that the intersection of red and green lines is produced around the threshold proposed by Dunn *et al.* [24]. Additionally, other studies performed on cerebrospinal fluid, serum or liver QC extracts also reported around 20% of CV on experimental replicates [25,26].

On the other hand, it is common that the nature of some biological samples and their limited availability complicates the analysis of QC samples. This was the case of mouse retinas in examples #1 and #2. Under these circumstances, there are not consensus standard criteria on how to handle analytical variation. We partially circumvent this issue using the following argument: Provided that the total variation of a metabolite feature (CV_T_) can be expressed as a sum of biological variation (CV_B_ ) and analytical variation (CV_A_) according to Equation (3), computed CV_T_ should be at minimum larger than 20% (the most accepted analytical variation threshold) for a metabolite feature to comprise biological variation.


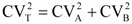
(3)

Therefore, when QC samples are not available we propose as rule of thumb to discard those features showing CV_T_ < 20% since biological variation is bellow analytical variation threshold. Figure 2 shows the frequency distribution of CV_T_ for working examples #1,2 and #4 where QC samples were not available. According to our criteria, those mzRT features to the left of the threshold will hold more analytical than biological variation and should be conveniently removed from further statistical analysis. This surely results in a too broad criterion since it assumes that the analytical variation of all metabolites is similar, which is of course not accurate given that instrumental drifts do not affect all metabolites evenly. It should be beard in mind, however, that tightly regulated metabolites presenting low variation such as glucose will likely be missed according to a 20% CV_T_ cut-off criterion. Of mention, example #2 and example #4 show the higher and lower percentage of mzRT features with more than 50% CV_T_ respectively. Therefore, there is more intrinsic variation in example #2 than in example#4. Whether such variation relates to the phenomena under study remain to be ascertained using hypothesis testing. 

## 5. Hypothesis Testing

Untargeted metabolomics studies focused in this paper are aimed at the discovery of those metabolites that are varied between two populations (*i.e.*, KO *vs* WT in examples #1 and 4 or treated *vs* untreated in example #3). In this sort of studies, random sample data from the populations to be compared are obtained in form of mzRT features dataset. Then, we calculate a statisti*c* value (usually *mean* or *median*) and use statistical inference to determine whether the observed differences in the median or mean of the two populations are due to the phenomena under study or to randomness. Statistical inference is the process of drawing statements or conclusions about a populations based on sample data in a way that the risk of error of such conclusions is specified. These conclusions are based on probabilities arisen from evidences given by sample data [27]. 

To characterize those varied mzRT features, data sets are usually specified via hypothesis testing. Conventionally, we first postulate a null difference between the means/median of metabolic features detected in the populations under study by setting a *null hypothesis* (H_0_). Then, we specify the probability threshold for this null hypothesis to be rejected when in fact it is true. This threshold of probability called α is frequently set-up at 5% and it can be though as the probability of a false positive result or *Type I error*. Then, we use hypothesis testing to calculate the probability (*p-value*) of null hypothesis rejection. Whenever this p-value is bellow to this pre-defined threshold of probability (α), we reject the null hypothesis. On the other hand, when calculated p-values are larger than α we do not have enough evidence to reject this hypothesis and we fail to reject it. Note that null hypothesis can never be proven, instead null hypothesis is either rejected or failed to reject. Conceptually, the failure to reject the null hypothesis (failure to find difference between the means) does not directly translate in to accept or prove it (showing that there is no difference in reality). 

A wide variety of univariate statistical tests to compare mean or medians are available. For a non-statistician it can be daunting to figure out which one is most appropriate to implement with an untargeted metabolomic design and dataset. Helpful guidelines in basic statistics books can be consulted [27,28]. As summarized in Table 2, two important considerations should be taken in to account when deciding for a particular test. First one is the experimental design and second one data distribution. 

**Table 2:** Best suited statistical tests for datasets following normal distribution or far from the normal curve according to their experimental design.

Experimental design	Normal distribution	Far from normal-curve
	Compare Means	Compare Medians
Compare two unpaired groups	Unpaired t-test	Mann-Whitney
Compare two paired groups	Paired t-test	Wilcoxon signed-rank
Compare more than two unmatched groups	One-way ANOVA with multiple comparison	Kruskal-Wallis
Compare more than two matched groups	Repeated-measures ANOVA	Friedman

Experimental design will depend on experimental conditions considered when the metabolomics study is designed. Once the experimental design is fixed, population distribution determines the type of the test. Depending on this distribution, there are essentially two families of tests: parametric and non-parametric. Parametric tests are based on the assumption that data are sampled from a Gaussian or normal distribution. Tests that do not make assumptions about the population distribution are referred as to non-parametric tests. Selection of parametric or non-parametric tests is not as clear-cut as might be a priori though. Next section deals with the calculations necessary to guide such decision and exemplifies these calculations with our four working examples. 

## 6. Deciding between Parametric or Non-Parametric Tests

### 6.1. Normality, Homogeneity of Variances and Independence Assumptions

Deciding between parametric and non-parametric tests should be based on three assumptions that should be checked: normality, homogeneity of variances (*i.e.*, homocedasticity) and independence. Nevertheless, some of these assumptions rely on very theoretical mathematical constructs hardly ever met by real-life datasets obtained from metabolomics experiments. 

Normality is assumed in parametric statistical tests such as t-test or ANOVA. Normal distributed populations are those presenting classical bell-shape curves to illustrate their probability density function. The frequency distribution of a normal population is a symmetric histogram with most of the frequency counts bunched in the middle and equally likely positive and negative deviations from this central value. The frequencies of these deviations fall off quickly as we move further away from this central point corresponding to the mean. Data sampled from normal populations can be fully characterized by just two parameters: the mean (m) and the standard deviation (σ). Normality assumption can be evaluated either statistically or graphically. We propose two tests to statistically evaluate normality: Shapiro-Wilk and Kolmogorov-Smirnov, the former better behaved in the case of small samples sizes (*i.e.,* N < 50) [27]. It is worth recalling that the term normal just applies to the entire population and not to the sample data. Hence, none of these tests would answer whether our dataset is normal or not. Their derived p-values must be interpreted as the probability of the data to be sampled from a normal distribution. On the other hand, testing normality is a matter of paradox: for small samples sizes normality tests lack from power to detect non-normal distributions and as sample size increases normality becomes less troublesome thanks to the Central Limit Theorem. Since parametric tests are robust again mild violations of normality (and equality of variances as well), the practice of preliminary testing these two assumptions has been regarded as setting out in a rowing boat in order to test whether it is safe to launch an ocean liner [29]. Additionally, normality tests can be complemented with descriptive statistics such as Skewness and Kurtosis. On the other hand, graphical methods such as histograms, probability plots or Q-Q plots might result also helpful as tools to evaluate normality. Their use, however, is rather limited at exploratory stage of LC-MS untargeted metabolomic data since it is unfeasible to examine each one of these plots for each mzRT feature detected. 

Another of the assumptions of a parametric test is that the within-group variances of the groups are all the same (exhibit homoscedasticity or homogeneity of variances). If the variances are different from each other (exhibit heteroscedasticity), the probability of obtaining a "significant" result even though the null hypothesis is true may be greater than the desired alpha level. There are both graphical and statistical methods for evaluating homoscedasticity. The graphical method is the so-called boxplot but again, its use is rather limited because the impossibility to evaluate each one of them separately. The statistical methods are Levene’s and Bartlett tests, the former the less sensitive to departures from normality. In both cases, the null hypothesis states that the group variances are equal. Resulting p-value < 0.05 indicate that the obtained differences in sample variances are unlikely to have occurred based on random sampling. Thus, the null hypothesis of equal variances is rejected and it is concluded that there is a difference between the population variances.

The third assumption refers to independence. Two events are independent when the occurrence of one event makes it neither more nor less probable that the other occurs. In our metabolomic context, the knowledge of the value of one sample entering the study provides no clue about the value of another sample to be drawn.

### 6.2. Parametric and Non-Parametric Tests. Does It Really Matters in LC-MS Untargeted Metabolomics Data?

Overall, the strength of violation of the three assumptions will determine the application of a parametric or non-parametric test. It should be noted that parametric tests are more powerful than non-parametric tests, *i.e.*, the use of a non-parametric test might miss a statistically significant difference that a parametric test would find. However, when dealing with non-normal populations, unequal variances, and unequal small sample sizes, a non-parametric test would perform better. This is the worst-case scenario for a parametric test to be non-robust. Although we recognize main weakness of normality testing, by way of example we have calculated the percentage of features that meet normality and homocedasticity assumptions in our four working examples (Table 3) 

**Table 3:** mzRT features percentages in which normality, homocedasticity or both assumptions are met. H_0_ (Shapiro-Wilk’s test)= Data are sampled from a Gaussian distribution. H_0_ (Levene’s test)=Variances are equal. Percentages represent those features in which there were not enough evidences to reject H_0_ at conventional α=0.05 relative to the total number of features retained after handling analytical variation.

	# mzRT	Groups	Normality (Shapiro-Wilk's test)	Homocedasticity (Levene’s test)	Normality & Homocedasticity
Example #1 (Retinas)	3252	KO	66%	93%	60%
		WT	60%		54%
Example #2 (Retinas)	7654	Normoxia	65%	77%	48%
		Hypoxia	79%		60%
Example #3 (Serum)	6131	Untreated	85%	90%	76%
		Treated	88%		78%
Example #4 (Neuronal cells)	6831	KO	72%	91%	64%
		WT	82%		73%

According to Table 3 and considering the four examples on average, 65% of detected features meet normality and equality of variances assumptions. Therefore the use of a parametric test would be acceptable in 65% of the cases. Using a parametric test on the entire dataset would result in lack of robustness and consequent inaccurate p-values for the remaining 35% of features that do not meet parametric test assumptions. Alternatively, considering the use of a non-parametric would turn in loss of statistical power for those 65% of features. Alternatively we would transform those non-normally distributed data to normal or near to normal, for example taking logarithms when data come from a lognormal distribution. Nevertheless, data transformation should be handled carefully since it might hamper the interpretation of the results. 

**Figure 3:**
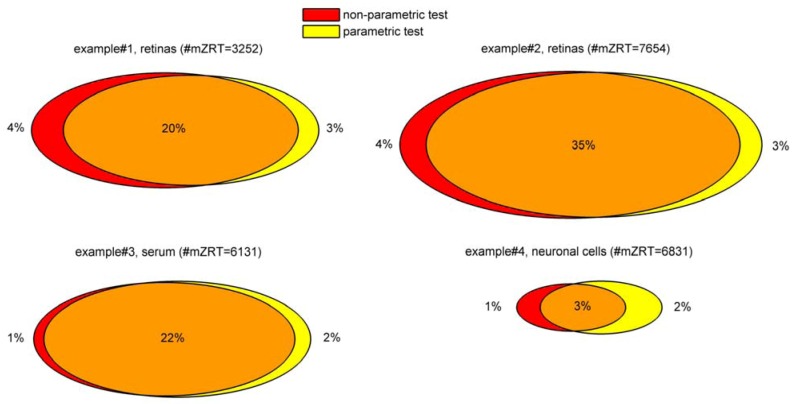
Venn-Diagrams of the mzRT features showing statistical significance using either parametric or non-parametric tests. Venn-Diagrams’ areas are proportional to the percentage of the significantly varied features out of the number of total features retained after handling analytical variation (indicated in parenthesis) .The Mann-Whitney test (examples #1, 2 and 4) or Wilcoxon signed rank (example #3) tests were used for non-parametric groups median comparisons. Unpaired (examples #1, 2 and 4) or paired (example #3) t-tests were used for parametric groups mean comparisons.

To evaluate the consequences of using parametric or non-parametric tests in our datasets, we performed both types of tests and compare their outcomes. The Venn diagrams in Figure 3 show the percentage of features resulting in significantly different means/medians using parametric and non-parametric tests for the four working examples. Both tests share most of the significantly varying features and just a minor percentage of the total were specifically detected using either parametric or non-parametric tests. In general terms, analysis on the four working examples show a residual discrepancy between parametric or non-parametric test in terms of their outlined significant features. Although from these results we can not extrapolate a general methodology to choose between parametric and non-parametric tests, we recommend testing normality and equality of variances assumptions prior hypothesis testing to gain deeper insights in population distributions. Then, performing both parametric and non-parametric tests and to compare their outcomes prevailing parametric test outcomes for further calculations. Notice that if parametric and non-parametric tests result in high discrepancy we should check for outliers in our dataset.

## 7. Using Multiple Related Tests that Cumulate the p-Value: The Multiple Testing Problem and the False Discovery Rate

### 7.1. The Multiple Testing Problem

In untargeted LC-MS-based metabolomics studies, the number of univariate-paralleled test equates to the number of mzRT features detected. As showed in our working examples, this number usually ranges in the thousands (it largely depends on experimental conditions). As the number of hypotheses tests increases, so as too does the probability of wrongly rejecting a null hypothesis because of random chance and therefore a substantial number of false positives (Type I error) might occur. This accumulation of false positives is termed the multiple testing problem and is a general property of a confidence-based statistical test when applied across multiple features. From a metabolomics research standpoint, Type I errors are particularly undesirable. A substantial amount of work and resources based on MS/MS confirmation experiment can be stimulated in favor of a false finding. In the worst case, a follow-up validation study on a false positive finding would not replicate the original work with consequent waste of resources and time. In such situations the chance for false positive rates must be carefully handed. Otherwise false findings may seriously affect the outcome of this type of studies [30]. Therefore, retrieved p-values from multiple tests performed in parallel across the detected mzRT features should be corrected. This is to re-calculate those probabilities obtained from a statistical test which is repeated multiple times. We are going to discuss two possible ways of handling multiple testing problem: the Bonferroni and the FDR (False discovery Rate) corrections.

### 7.2. Bonferroni Correction

The family wise error (FWER) is defined as the probability of yielding one or more false positives out of all hypotheses tested. This error remains the most accepted parameter for ascribing significance levels to statistical test [31,32]. In multiple testing, if k independent comparison are performed FWER is increased at the rate of 1-(1-α)^k^; where k is the number of hypothesis tests performed and α is the pre-defined threshold of probability in each individual test. Therefore, to maintain a prescribed FWER (*i.e.* 0.05) in an analysis involving multiple tests, the α assumed in each independent test must be more stringent than FWER. Bonferroni correction is the standard approach to control FWER by specifying what α values should be considered in each individual test using the Equation 4:


α = FWER/k
(4)

Considering our working example #1, 3252 mzRT features were retained after handling analytical variation. According to Bonferroni correction we should set a corrected α=0.05/3252=1.054×10^-5^ for each individual test to accept an overall FWER of 0.05. Hence, in each individual test, only those features with p-values ≤ 1.54 × 10^-5^ would be declared to be statistically significant. Assuming this correction, the probability of yielding one or more false positives out of all 3252 hypotheses tested would be FWER = 1-(1-1.54 × 10^-5^)^3252^ = 0.0488. Notice that this probability is much lower than the one obtained if no correction was applied: FWER = 1-(1-0.05)^4581^ ≈ 1. Bonferroni correction represents a substantial increase of the stringency of our testing leading to just 75 metabolite features out of the initially 3252 prescribing a FWER = 0.05.

Bonferroni correction keep a strict control on making one or more Type I error (false positive) at expenses of Type II errors (false negative). However, false negative findings might cause to overlook metabolites of potential interest and they also affect the outcomes of an untargeted metabolomics study. Other approaches to multiple testing correction such as the FDR (False Discovery Rate) claims for a striking balance between the concern about making too many false discoveries and the concern about missing the discovery of a real difference [33]. Next section deals on FDR correction and its interpretation.

### 7.3. The FDR Multiple Testing Correction

The FDR compute the number of false positives out of the significantly varied metabolic features, *i.e.*, the rate of significant features being false. This is different from the Bonferroni correction which focuses on the control on all falsely rejected hypotheses. In other fields such as microarray data experiments, the Bonferroni correction has been found to be too conservative and its use has led to many missed features of interest [33]. It has been argued that controlling the rate of allowed false findings using FDR do not represent a serious problem in the context of an exploratory research when further confirmatory studies are undertaken [31,32,33]. In addition, it has been demonstrated that controlling the FDR at the screening stage of the research carries a benefit for the next research stages [34]. Nevertheless, some authors in the field of metabolomics advocate that although being the most conservative, a Bonferroni analysis is both conceptually easier to understand and numerically easier to implement [35]. 

FDR correction calculates a p-corrected value or q-value for each tested metabolic feature. This q-value is a function of the p-values and the distribution of the entire set of p-values from the family of tests being considered [31]. For each feature, its associated q-value can be though as the expected proportion of false positives considered when such feature is declared to be significantly varied. Hence, a metabolic feature having a q-value of 0.05 implies that 5% of metabolic features showing p-values as small as such feature are false positives. A useful consideration is that a p-value of 0.05 implies that 5% of all tests will result in false positives and a q-value of 0.05 means that 5% out of the significant tests will result in false positives. 

**Figure 4:**
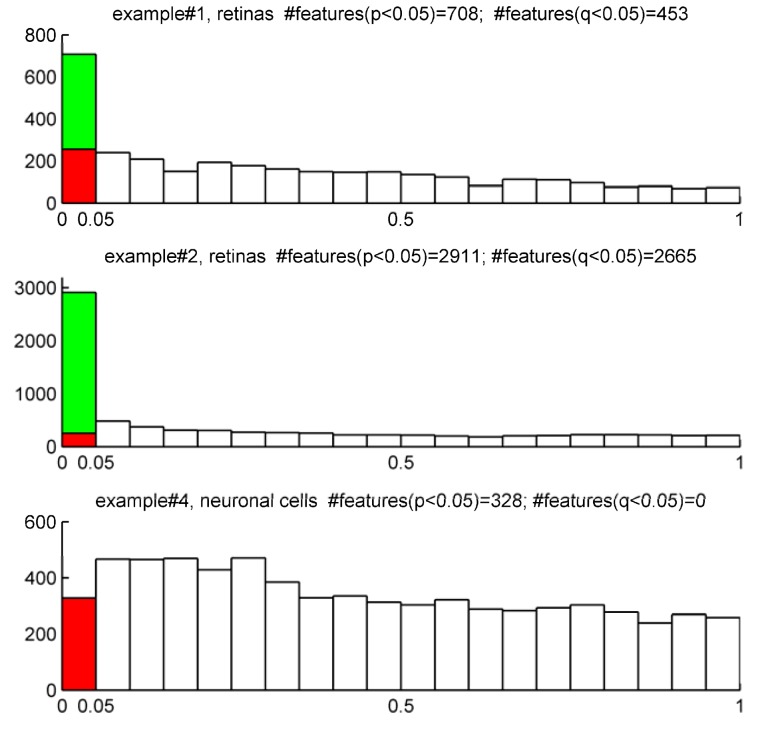
Frequency histogram showing the distribution of p-values typically expected from t-tests binary groups’ comparison in examples #1, 2 and 4. Green bar represent the total number of features declared to be significant assuming 5% false positives in a t-test comparison of the two groups. Red bar represent the FDR- estimated number of features being considered false positives out of the features declared significant in the t-test. The number of total significant features retained after FDR correction (q < 0.05) is also indicated.

A useful plot to evaluate the proportions of false positives is a frequency histogram illustrating the distribution of p-values obtained from paralleled tests across all mzRT features in a dataset. Figure 4 illustrates such histograms for examples #1, #2 and #4. Those mzRT features with significant changes in their relative abundance will show small p-values and therefore the histogram will be skewed towards 0 (examples #1 and 2). On the contrary, metabolic features showing no change in their relative abundances will show a uniform random flatten frequency distribution (example #4). The green bar represents those metabolic features declared to be significant in the t-test binary group comparison for each example (p < 0.05). The actual FDR calculated proportion of such features resulting in false positives correspond to the red bar (q-values > 0.05). 

According to Figure 4, t-test comparison of KO and WT groups in example#1 lead to 708 significantly changed metabolic features out of 3252. By setting our α threshold to 5% we accepted 163 features to be false positives. This represents 23% out of the 708 features significantly varied. Notice that after FDR correction we obtained 453 mzRT features with q-values bellow 5% of false positives acceptance threshold. This means that 5% out of this 453 mzRT features (*i.e.*, 23) are expected to be false positives. An acceptance of 5% chance of false positives results in a better situation than the one derived if no correction was applied (meaning 23% chance of false positives). Recall that in this same example, Bonferroni correction lead to consider just 75 features with an adjusted threshold p-value< 1.54×10^-5^. Bonferroni provides the strongest control of the false positives and therefore a high confidence in the selected metabolic features. However, an important advantage of FDR approach is that it allows the researcher to select the error rate that they would assume in their subsequent studies. On the other hand, Figure 4 show that a t-test comparison of WT and KO groups on example#4 outlined 328 features all of them resulting in false positives after FDR correction. This indicates that all this significant outcomes derived from chance and no real effect was underlying on this example. Accordingly if no correction for multiple testing were considered we would have done subsequent MS/MS identification experiments on features that represent false positives. This would have been a pointless task with consequent waste of time and resources. To avoid situations like this, we would recommend correcting for multiple testing when dealing with multiple univariate analysis of untargeted LC-MS datasets. Then, focus on those metabolites with lower FDR derived q-values for further MS/MS identification experiments. In addition, we would like to comment that whenever a follow-up targeted validation study was going to be attempted, we would recommend considering those metabolites showing statistical significance after strict Bonferroni correction.

## 8. The Fold Change Criteria

A common practice to identify mzRT features of relevance within a dataset is to rank these features according their fold change (FC). FC can be though as the magnitude of difference between the two populations under study. For each mzRT feature, a FC value is computed according to equation 5 in which X represents the average raw intensities across “case” group and Y represents the average raw intensities across “control” group. Whenever the raw intensities of the “control” group are larger than in the “case” group, this ratio should be inverted and sign should be conveniently changed to indicate a decrease of the case group relative to the control. Of mention, in paired-data designs, fold change should be calculated as the average of each individual fold change across all sample pairs.


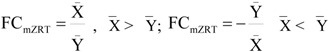
(5)

In formal statistical terms, a mzRT feature is claim to be varied among two conditions when its relative intensity values change systematically between these two condition regardless on how small this change is. However, significance does not contain information about the magnitude of this change. For a metabolomics standpoint, a metabolic feature is considered to be relevant only when this change result in a worthwhile amount. Hence, significantly varied mZRT are ranked according to their FC value. Subsequent MS/MS chemical structural identification experiments are performed on those metabolic features resulting above a minimum FC cutoff value. It has been demonstrated that a 2-FC cutoff for metabolomics studies using human plasma or CSF minimizes the effects of biological variation inherent in a healthy control group [26]. However, this cutoff value is set rather arbitrarily and based on similar FC cutoff values routinely applied in gene chip experiments.

## 9. Univariate LC-MS Untargeted Analysis Workflow

**Figure 5:**
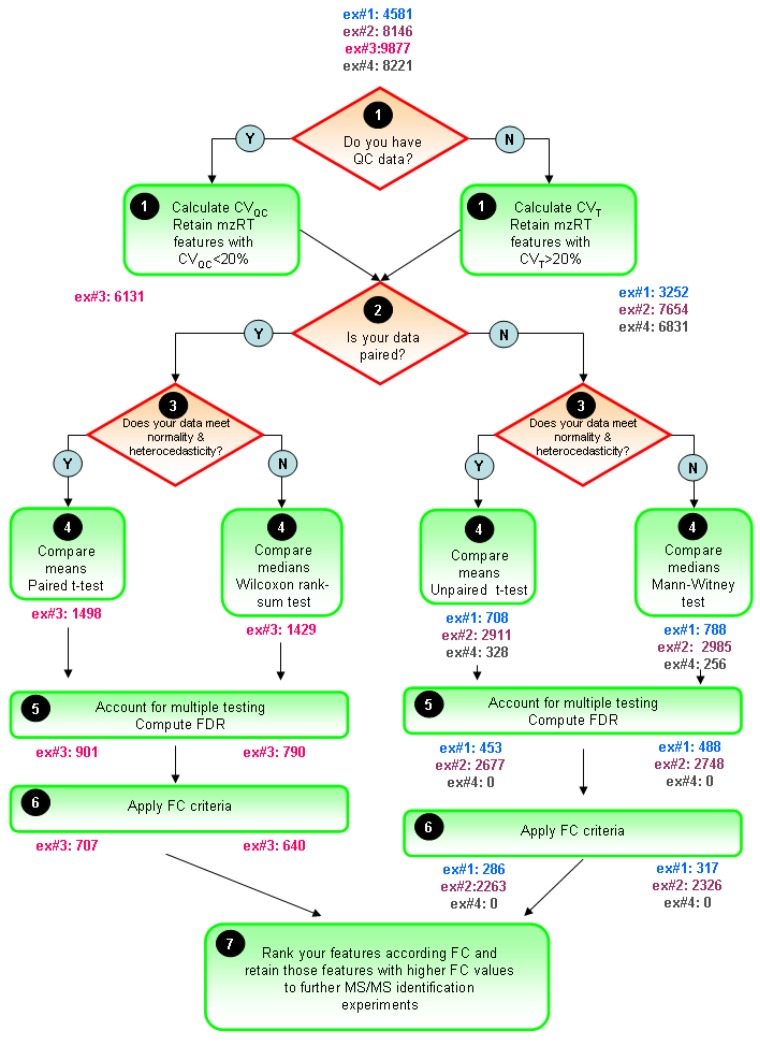
General flow chart for univariate data analysis of untargeted LC-MS-based metabolomics data. Different colors for the four working examples indicate the initial number and the retained number of mzRT features in each step. FDR and FC value are fixed at 5% level 1.5-cutoff values respectively.

The typical univariate data analysis flow diagram for untargeted LC-MS metabolomics experiments is summarized in Figure 5. The ultimate goal is to constraint the number of initially detected mzRT features to an amenable number for further MS/MS identification experiments. Only those mZRT features showing both statistically significant changes with delimited chance for false positives in their relative intensity and a minimum FC are going to be retained. Steps 1-5 are below summarized:

STEP1: Use quality control check to get rid-out of those mZRT features that do not contain biological information. Ideally QC samples should be measured. Then, compute CV_QC_ and proceed to retain only those metabolic features presenting CV_QC_ < 20%. If QC samples are not available, an alternative procedure is to compute CV_T_ and retain those mZRT with CV_T_ > 20%. 

STEP2: Mind the experimental design to select the best suited statistical test to apply. Check whether your data is paired or not, *i.e.*, whether your groups are related such as in our example#3 (individuals prior to treatment are uniquely matched to the same individual after the treatment). Afterwards, check normality and equality of variances assumptions. Be aware that performances of the normality tests might be hampered by low samples sizes dataset commonly found in LC-MS untargeted metabolomics studies. Despite this, working on such tests might be useful to gain some insights in to the data distribution. 

STEP 3: Compare mean or medians of your dataset performing statistical inference and trying to apply statistical tests thoughtfully instead of mechanically. Try to be aware of the tests weaknesses when applying it. Once we have taken the decision on whether using parametric or non-parametric tests, it is important to stick on the same approach through the rest of the data analysis procedure. This is to plot our results in the form of medians instead of means whenever we choose to use a non-parametric statistical test. 

STEP4: Account for multiple testing. Report the number of positive false findings after FDR correction. Plot histograms of p-values frequency distribution to get an overview of whether a dataset contains significant differences. Decide a FDR threshold to accept. A general consensus is to accept 5% of FDR level but there is nothing special about this value and each researcher might justify their assumed FDR value, which should be fixed before data is collected. 

STEP5: Compute mean or median FC depending on the test used to perform statistical inference. Fix a cutoff FC value. From our in-house experience we recommend an arbitrary 1.5-FC cutoff value meaning a minimum of 50% of variation in the two groups compared. Rank your significant list of features according the FC value. Retain those significant features with higher FC values for MS/MS experiments and follow-up validation studies.

Following steps 1-5 described above, those metabolites identified using MS/MS experiments for example #2 are summarized in Table 4. Of mention all metabolites identified meet the statistical criteria described above regardless of using either parametric or non-parametric tests. Notice the small number of properly identified metabolites as compared to the high number of features surviving statistical criteria. It is important to mention that in the best optimistic case the number of metabolite identifications showing MS/MS confirmation use to be in the tens after a formal untargeted metabolomics experiment. Conversely, in case of putative identifications based on exact masses, the number of metabolites reported is much higher. However, recall that such metabolites are just putatively identified. Considering that replication experiments are necessary to undeniably ascertain the role of the metabolites found to be relevant in the untargeted study, a strict identification of the metabolites is essential. In this sense, our work-flow data analysis represents the first step for a successful identification of those metabolites. 

**Table 4:** Statistics summary of those metabolites identified using MS/MS experiments in working example #2. Unpaired t-test and Mann-Whitney test were used for parametric and non-parametric hypoxic and normoxic retinas comparison respectively. Correction for multiple testing was performed assuming 5% FDR.

	Parametric Test			Non-parametric		
	p-value	q- value	FC (mean)	p-value	q-value	FC (median)
**Hexadecenoylcarnitine**	3.31×10^-13^	1.05×10^-10^	5.0	2.49×10^-05^	3.18×10^-04^	**4.9**
**Acetylcarnitine-derivative**	1.10×10^-13^	5.02×10^-11^	7.2	2.49×10^-05^	3.18×10^-04^	**7.5**
**Tetradecenoylcarnitine**	1.29×10^-13^	5.29×10^-11^	8.8	2.49×10^-05^	3.18×10^-04^	**8.8**
**Decanoylcarnitine**	7.79×10^-11^	1.03×10^-08^	5.7	2.49×10^-05^	3.18×10^-04^	**5.6**
**Laurylcarnitine**	8.48×10^-11^	1.06×10^-08^	9.2	2.49×10^-05^	3.18×10^-04^	**8.7**
**7-ketocholesterol**	4.00×10^-09^	1.92×10^-07^	3.1	2.49×10^-05^	3.18×10^-04^	**3.3**
**5,6β-epoxy-cholesterol**	2.12×10^-08^	6.61×10^-07^	5.1	2.49×10^-05^	3.18×10^-04^	**7.0**
**7α-hydroxycholesterol**	3.88×10^-08^	1.07×10^-06^	4.1	2.49×10^-05^	3.18×10^-04^	**4.5**
**All-trans-Retinal**	1.26×10^-05^	9.24×10^-05^	-3.0	4.01×10^-05^	3.98×10^-04^	**-2.8**
**Octanoylcarnitine**	**9.21×10^-05^**	**4.28×10^-04^**	**5.5**	**5.09×10^-03^**	**1.14×10^-02^**	**17.2**
